# Talking Dogs: The Paradoxes Inherent in the Cultural Phenomenon of Soundboard Use by Dogs

**DOI:** 10.3390/ani14223272

**Published:** 2024-11-14

**Authors:** Justyna Włodarczyk, Jack Harrison, Sara L. Kruszona-Barełkowska, Clive D. L. Wynne

**Affiliations:** 1 Institute of English Studies, University of Warsaw, 00-312 Warszawa, Poland; j.wlodarczyk@uw.edu.pl (J.W.); j.harrison@uw.edu.pl (J.H.); sara@kruszona.eu (S.L.K.-B.); 2 Department of Psychology, Arizona State University, Tempe, AZ 85287, USA

**Keywords:** talking dogs, human–animal relationship, social media, dog training, animal performances

## Abstract

In recent years, dogs that appear to communicate with people by pressing buttons on soundboards that replay pre-recorded English words have become very popular on social media online. We explore how these dogs belong to a historical tradition that dates back at least to the Middle Ages and peaked in the early twentieth century. Through analyses of short videos, books, and training manuals, we identify several paradoxes inherent in this phenomenon. These include how the dogs appear to provide unmediated access to their thoughts, and yet, their button presses are typically incoherent and require interpretation. They also require months of training to “spontaneously” express themselves. There is also an anthropomorphism and -centrism in claiming that a human skill—language—is required for a dog to express mental states that it already possesses. This approach to communicating with dogs quiets canine forms of expression such as barking, whining, bodily postures, and odors and replaces them with endearing but infantile human voices. We suggest that, while this endeavor may be well intentioned and often playful, it runs the risk of skewing people’s perception of dogs towards fur-clad infants rather than adult members of a different species and of making people less attentive to canine nonverbal communication.

## Simple Summary

Dogs that press buttons to communicate with people have attracted a lot of attention on social media recently. This paper explores how communicating with dogs has a long history—particularly as entertainment but also as science—and notes several paradoxes in the presentation of modern button-pressing dogs. These include how these animals are presented as spontaneously expressing their thoughts when they need months of training; how they appear to offer direct access to their thoughts, yet their button presses usually make little sense without someone interpreting them; and how a human skill—language—is needed for dogs to express their canine mental states. We conclude that although well intentioned and playful, this approach to communicating with dogs runs the risk of replacing the ways that dogs truly communicate with people, such as barking and whining, with an infantile form of human communication.

## 1. Introduction

### 1.1. Soundboard-Using Dogs

“Dog Why?”, “Dog What Dog Is?”—these are quotes from Bunny, a Sheepadoodle known for using speech buttons on a soundboard to communicate with humans. Bunny’s TikTok account, wherein her owner regularly shares videos of their “conversations”, currently has 8.6 million followers. Many similar accounts also generate millions of views, highlighting the internet’s fascination with “talking dog” videos. This phenomenon is recent, originating with media coverage of speech therapist Christina Hunger’s blog, hungerforwords.com, in November 2019 [1], and the publication of Hunger’s book in 2021. The popularity of these videos has prompted an influx of related content, including memoirs by pet guardians, training manuals, and a proliferation of media articles and videos discussing the phenomenon as well as sales of the speech buttons themselves.

This form of human–canine communication is marketed as a new step in enriching the bond between people and dogs, claiming to give dogs the ability to communicate their needs directly to their owners through technology. For example, the advertising for FluentPet, the brand of soundboard associated with Bunny and Alexis Devine, claims, “Every dog and cat can learn to communicate with buttons” [2]. Bunny’s videos are particularly intriguing and controversial because they suggest not only that dogs can master the use of language but that doing so might enhance their self-awareness and agency.

Researchers have begun addressing the various ways novel technologies can be used to aid human–animal communication [3], and the first studies have been published specifically on soundboard-using dogs [4,5]. The goal of this article is not to determine whether dogs can internalize grammatical structures or use language creatively. Rather, we analyze the phenomenon of soundboard-using pets from a different perspective: a cultural–historical one. This article examines the “talking dogs” trend as a cultural phenomenon, highlighting the paradoxes within the assumptions of the proponents of augmentative interspecies communication devices [6]. By analyzing the cultural products associated with soundboards for pets (videos posted to social media, trainers’ memoirs, and manuals), we explore how this trend is both novel—enabled by contemporary internet culture and the availability of inexpensive sound playback devices—and shaped by a long history of anthropomorphized canine performances, where dogs imitate human form and behavior to elicit laughter from a human audience.

We also assess how the cultural products associated with soundboards fit into broader paradigms of understanding human–canine relationships. However, after analyzing the videos and popular literature surrounding this trend, we propose some alternative explanations for why dogs could be pressing soundboard buttons. We also identify a tension between respecting canine uniqueness and the imposition of a human-designed communication technology.

On its surface, the use of augmentative interspecies communication devices seems to align with a broader shift towards recognizing and respecting canine specificity and improving canine welfare, a trend noted by many scholars studying shifts in the human–canine bond. After all, these buttons have the potential to enable dogs to “tell” their owners about their needs and desires. However, this broader movement also emphasizes the importance of canine-specific modes of communication, which soundboards do not accommodate. The paradox is rooted in the erroneous assumption that while dogs possess rich internal mental states, they lack adequate channels to express them. This assumption stems from an anthropocentric worldview that privileges speech as the primary mode of communication and measures intelligence through skillful language use. To address this, we must begin by drafting a working distinction between “communication” and “language”.

### 1.2. Definitions

Communication is a process by which one individual (a sender) sends a signal to another (the receiver), where signals are behaviors specifically evolved to provide information [7]. Communication is a commonplace in the animal kingdom. Many species have evolved species-specific forms of communication that enable the transfer of information between conspecifics such as the “dance language” by which foraging honeybees communicate the location of the nectar they have discovered to their hive-mates [8].

“Language” will be used here to distinguish communication systems that share crucial features with natural human languages. The specific features of human language that differentiate it from most forms of communication in other species remain controversial (see, e.g., [9]), but it shows a level of complexity and flexibility not seen in other species [7] as well as a hierarchical syntactic structure [9]. Although most natural languages are vocal, it is not essential that language be communicated through auditory channels. Hunger and other proponents of augmented interspecies communication refer to the communicative skills they ‘install’ in their dogs as “talk”, implying that they see dogs as capable not only of communication but of language.

Most proponents of communication with dogs through soundboards do not perceive the activity they are engaging in with their dogs as training. Teaching dogs to use a soundboard is seen as providing them with tools that allow them to express their otherwise ineffable mental states. In everyday English, “training” implies the intentional imposition by one individual of a program of discipline or instruction on another in order to change their behavior [10]. One of the insights of behavioral psychology is that individuals may become trained (their behavior becomes more adept) without the need for conscious application of any instruction by another. Behavior followed by positive consequences is more likely to be repeated in the future; that which is followed by unattractive consequences is less likely to be repeated [11], and similarly, initially unimportant stimuli may come to alter behavior if they are reliably followed by important consequences [12]. Since the application of these stimuli and consequences can follow unconsciously or even be provided by inanimate objects (as when a stove burns the finger of a too-inquisitive child), it follows that there is no need for an individual to intentionally act as a “trainer” for another individual to receive “training”.

Many contemporary canine enthusiasts associate the term “training” with the imposition of human control on a dog’s behavior and with the performance of behaviors on cue [13]. Consequently, many contemporary dog owners, interested more in developing interspecies bonds than in the dog’s performance of specific actions, resist the word training and prefer to refer to their interactions with dogs as socialization, behavior modification, the teaching of life skills, etc. [13]. Hunger, in her book on the use of soundboards with dogs, also refrains from using the word training. However, if the definitions from the previous paragraph are applied, training can be viewed as a much broader endeavor than simply the conscious teaching of behaviors to be performed on cue. In other words, training may be taking place even if the dogs’ owners are not consciously training the dogs.

## 2. History of Talking Animals: Entertainment and Science

### 2.1. Medieval and Earlier Performing Dogs

Although the technology enabling the phenomenon of contemporary “talking dogs” is relatively recent, animal trainers have for centuries been crafting performances in which dogs appear to communicate with humans in various ways. However, the goal of these activities was—with few exceptions—not scientific investigation into language acquisition but the creation of an entertaining illusion. The popularity of these historical performances seems to have been grounded in large part in anthropomorphism, which links these dog trick acts with contemporary soundboard-using dogs.

The history of teaching dogs to appear to speak is inseparable from the history of popular performances of animal sagacity and dates back at least to the Middle Ages [14]. It is a history in which canine abilities have evoked responses of awe, disbelief, and laughter, responses in which the possibility of the audience being tricked by the dog’s performance has always been in the background [13]. The history of canine performances as entertainment undoubtedly influences the current perception of the phenomenon of dogs using soundboards. Modern online audiences may be drawn to these videos for reasons similar to those that drew audiences to purchase tickets for trick dog performances in the past.

In the Middle Ages, dogs were common animal performers, due to their availability and trainability [14,15]. Writing about animal acts in the period 1000–1400 in Western Europe, Lisa Kiser mentions a weeping dog and a dog that could “imitate human actions on command” and that “makes the king laugh” [14] (p. 121). Kiser infers that almost all of the acts that have left evidence of their content featured animals engaging in human-like behaviors [14]. This suggests that medieval audiences considered the breaking of the usual boundary between animals and humans to be the most entertaining type of animal performance. Such performances relied on anthropomorphism, thereby replicating patterns known to the audience from popular animal fables dating back to ancient Greece [15,16] and one which influences literary and visual representations of talking animals to this day [17,18].

Although the imitation by dogs of human behavior and language remained the most popular form of canine-related entertainment throughout the medieval and early-modern period [13,19,20], the appearance of speech could also arouse suspicions of demonic possession of the animal and its owner. In fact, during the first witchcraft trial in England in 1566, the accusations against Agnes Waterhouse were supported by her neighbor’s recollection of the accused woman’s dog visiting the neighbor and asking for some butter [21,22]. This tradition does not have a strong presence in contemporary culture, where fear-inducing, speaking animals are confined to horror movies.

The written record of “sagacious dogs” in Europe and North America becomes more abundant in the eighteenth and nineteenth centuries. For example, eighteenth-century Paris hosted multiple performing dogs, including, “a little dog that was seen reading French and English and performing numerous other tricks” [21] (p. 13). It seems likely that the trainers were very aware of the fact that the dogs’ performances of intelligence were carefully crafted acts.

### 2.2. Victorian Talking Dogs: Entertainment and Science

Two nineteenth-century attempts to test the limits of language acquisition by canines stand out for utilizing a more scientific approach. These were carried out in the 1860s by Alexander Graham Bell, the inventor of the telephone, and by Sir John Lubbock, a banker, politician, and polymath and a friend and neighbor of Charles Darwin, in the 1880s. Bell shaped the mouth of his terrier mix, Trouve, through touch stimulation in order to elicit sounds. Bell was able to elicit the words “mamma” and “grandmamma” from the dog but could not proceed further [23]. Bell’s experiments with Trouve strongly suggested that vocalization by canines was, if not totally impossible, at least very difficult.

Lubbock’s attempt to teach language to his terrier, Van, also provides a more detailed and scientific reflection on animals’ capabilities for language. Lubbock [24] trained his dog to associate cards with words written on them with actions and thereby make simple requests (e.g., “Out”, “Bone”, and “Water”) by bringing the cards to the handler. Lubbock also describes his lack of success in teaching Van to distinguish colors and to count and acknowledges finding inspiration in a method devised to work with deaf and blind children. In other words, Lubbock saw his system in which the dog makes requests using a technologically mediated version of human speech not as a party trick but as a translation strategy to bridge the gap between animal and human minds by giving the dog tools to communicate his needs and desires. Lubbock also recognized that dogs can have desires of their own and are capable of communicating them to their owners. As summed up by [23] (p. 165), “The watershed that Lubbock and Van provide is between the belief that animals enjoy some kind of mentation, perhaps consciousness, some ability to reason and feel—and that with appropriate techniques, human beings can come to understand and appreciate the mentality of animal life”. However, unlike current “talking dog” trainers, Lubbock never claimed dogs were capable of abstract thought and of spontaneously producing grammatical multi-word utterances.

### 2.3. Twentieth-Century Clever Dogs

Staged performances of canine sagacity increased in popularity after the career of Clever Hans, a German horse at the beginning of the twentieth century who appeared capable of advanced math and other cognitive feats [25]. Imitators of Clever Hans were keen to work with the species closest to humans and easiest to source: the dog. Among the most famous of these talking dogs was the German Airedale terrier Rolf, who could count and tap out complex and grammatically correct messages using a symbolic representation of the alphabet [26]. Rolf’s owner Paula Moekel recounted the story of her wonderdog and his progeny in her book, *Mein Hund Rolf* [My Dog Rolf] (1919) [27]. This first-person account set the pattern for subsequent books by the owners of talking dogs.

In the early 1960s, Elizabeth Mann Borgese, daughter of writer Thomas Mann, taught her English setter Arli to type out words on a modified typewriter. Borgese’s ideological grounding of the project and her training methodology are described in her book *The Language Barrier: Beasts and Men* (1965) [28]. Borgese uses the language of “uplift” to explain her endeavor, suggesting that providing animals with a channel through which they could communicate with humans is both akin to erasing the assumed disability of speechlessness and simultaneously a form of uplifting their minds to a higher level. Borgese concludes that learning human speech is not just a channel of expression but one of development: “It [language learning] is a dynamic process. It develops the brain potentialities of the experimental animal” [28] (p. 5). Just like Lubbock and Bell, Borgese posits an analogy between disabled humans—the deaf and the blind—and nonhuman animals: they both lack a voice and this deficiency can be overcome through the application of training methods utilizing technological devices.

Borgese began to teach Arli to type out words on a modified typewriter by associating a reward with the action of pressing a letter key. She was quickly able to postpone the reward until the dog had typed out an entire word. However, as Arli progressed, she became increasingly aware that he was not actually expressing himself but performing a trained behavior. The most interesting moment in her account is when the dog, who is suffering from a stomachache, types out “a bad a bad doog” [28] (p. 60). This makes Borgese wonder whether Arli has hit on the sequence by chance, as these were words she asked him to type out quite often, or whether his phrasing was an intentional choice. She concludes, “Had he hit on the sequence ‘Arli go bed’ or ‘Arli go car’, it would have looked just as pathetic or just as cute” [28] (p. 60). Thus, Borgese acknowledges the possibility that the sequence of key presses by the dog was random. It appears possible that Arli had learnt to produce multi-word utterances by associating a reward with multiple button presses, a possibility we consider may also be the case with contemporary “talking dogs”.

Borgese mainly practiced dictation with Arli, but she also allowed the setter to express himself without instruction. In this exercise, the dog produced complex utterances that consisted of gibberish interspersed with some recognizable words. It seems that, at this stage, Borgese became convinced that the dog’s writing was nothing more than a party trick and, in a tongue-in-cheek manner, recalls an exchange of correspondence that she had with a “well-known critic of modern poetry” about the possible interpretations of Arli’s “poems” [28] (p. 63). However, the exercise of free-typing is a stressful one for Arli: “Spontaneous typing is hard on Arli’s nerves. He gets restless, He begins to hit the keys with his paw. He begins to whine and whimper and yelp” [28] (p. 64). Rather than rethinking the ideological grounding of her project and her training methodology, Borgese became convinced that dogs are capable only of following human instruction, rather than of communicating spontaneously.

These texts by Moekel and Borgese provide detailed accounts of both the teaching methods and the ideological grounding of these projects to teach dogs human language. However, they must be approached carefully because they only present the owner’s/writer’s subjective perspective. For example, Moekel’s account is clearly aimed at convincing the reader of the genuineness of Rolf’s amazing abilities, and this is a position from which she benefits as the owner of the famous dog. Moekel acts as the dog’s advocate and—more importantly—translator. Rolf’s communications can never be directly understood by the public and Moekel’s presence is necessary for the act to become intelligible to outsiders. This role of the dog’s owner as interpreter of its button presses is also visible in the books and video clips by the contemporary authors/trainers analyzed below.

Few of the attempts described above were carried out with the goal of genuinely researching dogs’ capability for language acquisition, and most attempts at canine language acquisition aimed, instead, to trick or entertain non-discerning audiences. The anthropomorphism of the performing dogs also evoked laughter: the imitation of human form by animals has always been closely allied with satire [29,30], and dogs, because of their availability and trainability, have been frequent contributors to such acts.

## 3. Talking Dogs: The Present-Day Phenomenon

### 3.1. The Videos

Contemporary talking dogs, using soundboards, are largely presented in short video clips posted on the social media platforms TikTok, YouTube, and Instagram. The clips that receive the highest numbers of views—and, consequently, the most revenue for their creators—are those in which the animals appear to express complex and abstract ideas, fluently engaging in longer conversations with their guardians, or using language in a way that creates a humorous effect. Although these videos appear to capture the animals’ spontaneous behavior, they often undergo significant editing to enhance their appeal.

The editing of the videos provides insights into the cultural factors that account for their popularity and reveals the efforts of the human video director. These edits include the addition of soundtracks, special effects, filters, and subtitles [31]. In these videos, the creators strive to keep the dog in the center of the frame, minimizing their own appearance, so that the dog is presented as the star of the video, narrating their lives.

Typically, these videos do not show the entire silhouette of the humans involved because the handler is usually holding the camera. Viewers might catch glimpses of arms or legs pressing buttons, but the focus remains on the dog. The handler’s voice usually comes from off-screen, and many recordings include a transcription of the conversation overlaid on the video, making it easier to follow. The main visual elements in these videos are the dog and the soundboard. This setup allows viewers to watch the dog pressing buttons, reacting to the handler’s statements, and awaiting a response. The viewer may get the impression that the dog is looking directly at the camera when it is really looking at the handler standing behind the camera. When there is a long pause as the dog considers its response, editing or time-lapse techniques are sometimes used to maintain the conversation’s pace and hide any behavior that might suggest the dog is hesitant. Such hesitation could undermine the portrayal of the dog as an intelligent, confident animal that understands the communication capabilities of the soundboard.

The videos shared on social media are edited so that viewers do not see the full context. Viewers are unaware of what happened before the conversation with the dog began or how it ultimately concluded and thus only receive a glimpse into the life of the human–animal pair. This is inevitable because the soundboard is confined to a particular space. While the depicted conversations appear clear and logical, viewers cannot be certain if this reflects the entire communication process or is a carefully curated selection of the more successful moments from longer, less coherent interactions. The final dialogue often resembles a somewhat disjointed and arrhythmic conversation with the dog using pre-recorded phrases, creating a slightly surreal yet endearing effect. The strong contextualization of the video by the owner also makes it impossible to determine the spontaneity of the dogs’ behavior.

A good example of this type of video is one shared by the TikTok account thechattylab. This popular channel, with 1.1 million followers, presents conversations with a female labrador, Copper, whose main interests, at least as presented in these videos, appear to be eating and playing in water. One video, titled “Pool….mad”, presents a story of the dog being upset about losing access to her pool. It opens with a shot of Copper calmly sitting and looking directly at the camera. Off-screen, the trainer’s voice, accompanied by a transcription, asks, “Hey Cop, what do you want?” The conversation begins as the dog sluggishly presses the “Pool” button. The trainer responds, “I know. Your pool is broken. I’m so sorry”. Throughout the conversation, the trainer expresses sympathy for the dog, occasionally using her hand or leg to press buttons herself. At one point, she asks, “Copper, want to play?” The dog presses the “Mad” button and looks away from the camera. The trainer again expresses sympathy and reiterates that the pool is broken. The video ends there, leaving viewers uncertain about how the conversation continued and concluded.

It is important to note here that the narrative is shaped by the human trainer; without the trainer’s commentary, viewers would not understand the connection between the “Pool” and “Mad” buttons. The trainer explains that the pool is broken, but it is hard to identify any emotions in the dog from the video snippet. The only reason to perceive the dog as “mad” would seem to be her selection of the “Mad” button: otherwise, her steady posture and slow movements do not convey a sense of anger or even agitation. She averts her gaze at the end of the video, but this might simply reflect discomfort with the camera being too close. Thus, there is a discrepancy between the dog’s body language and the narrative presented in the video. The dog’s “mad” state is a story skillfully imposed on the viewer through the handler’s choice of words. Additionally, the trainer’s hand and leg pressing the buttons serve as tools to guide the situation by encouraging the dog to interact with the soundboard. In most of the training protocols discussed below (Section 3.2 and Section 3.3), dogs are not forced to use the buttons, nor are they rewarded with food, but they are encouraged by the handler’s actions. Research has shown that dogs respond to human body language, verbal commands, and attentional cues [32,33,34], are sensitive to human social stimuli, and can use cues from human actions, with their responses depending on the context and their prior experiences [35]. In this video, the dog does not appear eager to interact with the soundboard. However, the human’s manipulations of the buttons may encourage the rather reluctant dog to engage in button pressing as does the location of the buttons: both “Pool” and “Mad” are at the edge of the large soundboard, closest to the dog’s location at the start of the video.

While we are not suggesting that all currently popular videos of dogs interacting with these soundboards are intentional manipulations, many employ editing techniques that heighten the effect of the dog’s interaction with the soundboard and that impose meaning on the dog’s otherwise ambiguous button presses.

### 3.2. Talking Dog Books: How Stella Learned to Talk and I Am Bunny

Even though public awareness of the phenomenon of dogs using soundboards has spread largely through short videos posted on TikTok and YouTube, two of the best-known human figures associated with the phenomenon of speech buttons, Christina Hunger and Alexis Devine, have published memoirs in which they discuss their motivations for teaching their dogs to use speech, trace the teaching process, and reflect on its results. Unlike the entertaining and short videos, the books devote more time and space to the perspective of the humans, who are largely absent from the videos. Both books, despite the different perspectives of their authors, exhibit the paradox that we have identified as underlying the phenomenon of “talking dogs”: the simultaneous recognition of and respect for canine otherness and a privileging of human speech as a preferred mode of communication.

Hunger [36] is a speech pathologist, and it is from this professional identity that she draws inspiration for her project of teaching Stella to use speech buttons, as she recounts in *How Stella Learned to Talk: The Groundbreaking Story of the World’s First Talking Dog* (2021). Even though she has no dog training experience, Hunger’s professional position increases the credibility of her narrative, which is permeated both with the vocabulary and underlying assumptions of human speech therapy—for example, Stella is referred to as a learner, just like speech-impaired children are in speech therapy textbooks. Hunger is motivated by her strong beliefs in the ideals of speech therapy—including respect for the learners and a presumption of their mental competence and desire to communicate. The guiding premise in her undertaking is the analogy between the learning potential of a speech-impaired child and a dog. Although this is never stated outright, Hunger’s method implies that the absence of a vocal apparatus in dogs is a pathology that can be corrected.

Hunger’s method for teaching dogs to use speech buttons is an almost exact replica of the methods she previously used with children, with the exception that physical plastic buttons replace icons on a touchscreen. Hunger often repeats the words she wants Stella to learn while modeling the activity or object she wants to associate them with; for example, when Stella was a puppy, Hunger would repeat the word “outside” while taking Stella out to the yard. It is Hunger’s reliance on human speech therapy models that makes her wary of the word “training”, which she associates only with the mechanical performance of behaviors on cue.

Hunger’s belief in the parallels between children’s and dogs’ language development is almost absolute, and this is why she hopes that the techniques she uses will eventually result in Stella’s spontaneous language production. She does not, however, reflect on the need for teaching human speech as a technology enabling communication with dogs. Within the world of speech therapy, speech is obviously the privileged and desired mode of communication. Hunger [36] (p. 40) recognizes speech-impaired children’s ability to communicate nonverbally and evaluates it as “a significant milestone on the way to words”. Nonverbal communication is still communication, but using speech is superior. Similarly, the use of soundboards by dogs can be summed up as the next stage in the development of the human–canine relationship. This seems so obvious to Hunger that it does not require further consideration.

Alexis Devine does not present herself as an expert in speech therapy or canine cognition—in fact, she is a first-time dog owner—but it is Devine [37], in *I Am Bunny: How a “Talking Dog” Taught Me Everything I Need to Know About Being Human*, who reflects in more depth on the ethics of imposing a human-centered mode of communication on animals. Conscious of the ethical pitfalls of language research with primates and of dogs’ ability to communicate nonverbally, she decides soundboards could serve as an additional channel of communication, one that does not impact canines’ nonverbal skills but that opens up new possibilities for dogs. Here, soundboards are linked with a supposed increase in canine agency. Likewise, even though Hunger does not reflect on the full repertoire of canine nonverbal communication, she also briefly contends that “Stella still acts like a dog, and communicates like a dog. The only difference is that she has one more tool to express herself” [36] (p. 150).

Hunger’s insistence on teaching Stella to produce multi-word utterances is understandable given that she is modeling her technique on children’s speech therapy methods. After all, the ultimate goal of speech therapy in children is not the uttering of single words but of sentences. However, Devine, who does not draw the parallel between children and dogs that is central to Hunger’s narrative, also insists on eliciting multi-word utterances from Bunny, preferably ones with correct tense markers. For example, Devine notes that when Bunny wants to go to the beach, she simply presses the button “Beach”, while Devine would prefer for Bunny to press a longer sequence of buttons that forms a sentence. Devine discusses what she does when this happens but does not reflect on why she takes on this course of action:

If she presses “Beach”, I’ll join her at the board and model “Bunny Want Beach” or another similar phrase. Sometimes when she presses “Beach” and I’d like a bit more exploration, I’ll remain silent, as I might in a shaping exercise, which may nudge her to repeat or elongate. Whatever her next utterance is, I’ll reward contextually for her effort so she doesn’t get so frustrated she stops trying to communicate

[37] (p. 84).

Interestingly, although Devine does not see her attempts to teach Bunny to speak as dog training, she does employ the vocabulary and the methods of a dog trainer here: avoiding frustration is a common concern among trainers who utilize shaping as a training method.

Despite their different backgrounds, both Hunger and Devine [36,37] appear convinced that their dogs are communicating with them and that they do so because they want to be understood: “What I found was that even without being reinforced with treats, the buttons were their own reward for Bunny. With this type of training, her primary reinforcer had become *being understood*” [37] (pp. 25–26, emphasis in original). In fact, Devine [37] (p. 103) evaluates the pushback against soundboard-using dogs as linked to the ambivalence generated by the idea of animal consciousness, namely, the fear that dogs may be sentient and may have internal lives similar to those of humans: “It seems a fear born entirely out of ego. A human inability to release a long-held, but inaccurate, belief that humans are the intellectually, morally, and emotionally superior species…. [A]nimal consciousness makes people uncomfortable”. Within this framework, soundboards become a tool for proving animal consciousness. There is, of course, a paradox inherent to this approach: the belief that only the acquisition of a human skill, that is, of language, can serve as proof of the mental powers that predate the skill.

While both books provide the motivation behind the humans’ involvement in soundboards and a description of the training process that is missing from the videos, it is important to remember that a certain form of self-fashioning of the author’s perspective always takes place in memoirs [38]. In Devine’s book, there is a striking absence of any discussion of Bunny’s social media accounts: while there is a mention of one of the viral videos, there is no self-reflective commentary on the link between Bunny’s language use and Devine’ social media career, even though the book delves into other aspects of the author’s personal life and background.

From a more theoretical perspective, it is significant that neither book emphasizes the exceptional intelligence of either Bunny or Stella. Unlike the earlier books by Moekel and Borgese, in which the speaking animals were lauded as uniquely gifted representatives of their species, Stella and Bunny are presented as dogs that have become able to express themselves as a result of being presented with the right tools. The possibility of any dog replicating these accomplishments is part of the appeal of these narratives.

### 3.3. Training Manuals and Training

While Hunger’s and Devine’s books focus more on the individual stories of the canine guardians and their dogs, there are also training manuals that provide step-by-step instructions for canine owners who wish to replicate Hunger’s and Devine’s results. These include Stephanie Rocha’s *Teach Your Dog to Talk* [39] and the *Fluentpet Starter Kit* [2], a booklet that accompanies the buttons that can be purchased from Fluentpet.com. These manuals, much more so than the videos and memoirs, provide insight into the methods used to train the dogs. While the booklets use the word teach rather than train, we use the word training in its broader meaning described in the introductory section.

Training an individual to emit a behavior through the provision of a desirable consequence is known as operant or instrumental conditioning [40,41]. A learner will learn more rapidly to produce different behaviors if those behaviors lead to different desired outcomes than if the behaviors produce the same outcome (the “differential outcomes effect” [42]). Consequently, the program that Lubbock [24] outlined, in which his dog learned to pick up cards with consequences written on them, with each card leading to a distinct outcome, would not be challenging to implement. However, if one outcome is clearly preferable to the dog over others, the result would likely mirror Lubbock’s experience; that is, a dog would probably select one or a small number of options that led to the most desired consequences.

Both sets of instructions for training dogs to operate the speech buttons outline methods similar to Lubbock’s [24], at least for initial training. The teacher first records themselves saying a word into the approximately 3 cm diameter button. Each subsequent button press causes the recorded word to be replayed. When the dog presses a button, it receives the desired consequence identified by the word the owner has recorded into it [2,39]. Interestingly, the instructions discourage the use of consumable rewards in favor of non-satiating outcomes like “Out” (access to the outside world) and “Play”, which reduce the risk of replicating Lubbock’s experience of a dog that tends to monopolize responses producing the most highly desired outcomes. A “No” button is also recommended to communicate that an action should be terminated.

How then to get from single button-press responses to more interesting multiple-button-press utterances? Both Fluentpet [2] (p. 18) and Rocha [39] emphasize praising the dog for any and all interactions with the buttons and then modeling the desired behavior by producing simple short sequences of button presses oneself. Fluentpet [2] (p. 18), for example, advises, “If they’re using one word button at a time, use one- or two-word buttons when you model. When your learner is using two different buttons in sequence, then you can go up to three words”. The implication of this is that, if the dog is sufficiently exposed to multi-word utterances, it will directly comprehend what is expected and start to “talk” in this form.

An alternative possibility is that the dogs in these training programs are responding to the rewards they receive for interacting with a number of buttons. Alan Neuringer and colleagues (e.g., [43,44]) demonstrated that diverse patterns of responding across a limited set of alternatives can be created if the trainer reinforces sequences of behavior that differ from the most recently produced patterns. In these experiments, pigeons were offered two keys they could peck: one on their left (L); another on their right (R). This could be analogous to a dog with two buttons. The birds were reinforced for series of pecks on either key. For example, they might be required to peck four times, leading to 16 (2^4^) possible sequences of left and right pecks. Neuringer only rewarded L and R patterns of pecks that had not occurred in some previous number of opportunities. For example, the pigeons might only be reinforced if the pattern of responses across the L and R keys had not been seen in the last five peck sequences. The pigeons were readily able to respond to these contingencies and produced highly variable patterns across the two keys. Although no similar studies have been reported for dogs, a particularly relevant study was carried out with songbirds that were successfully reinforced for producing diverse songs [45]. There is no reason to believe that this kind of procedure would not be successful in dogs and much of the description of how dogs are encouraged to press multiple buttons seems to fit this pattern of rewarding novel patterns of button presses.

None of the published reports on talking dogs are entirely explicit about the training regimen their dogs were exposed to. Both Hunger and Devine (as well as Fluentpet and Rocha [2,36,37,39]) discourage the use of consumable rewards, but of course, dogs’ behavior can be reinforced in many other ways than just with food. Many buttons lead to consequences that are desirable for a dog, such as playing or going outside, but the praise that owners are encouraged to give their dogs for interacting with the soundboard is itself reinforcing for these social animals [46]. It is therefore very plausible that the kind of contingencies Neuringer and colleagues outlined as effective in leading to animals that will produce interestingly varied patterns of behavior are in place when people work with button-pressing dogs.

### 3.4. Talking: Strengthening or Effacing Canine Voice?

When a dog presses a button that activates a recorded voice, the voice we hear is human. However, “voice” does not only refer to vocal sounds but also the performance of identity and the agency to make things happen [47]. Voice in this sense is an important concept within animal rights discourse. Animals are often framed as lacking a voice that could enable them to speak up about their abuse and exploitation at the hands of human beings [48]. In this sense, animals lack a voice precisely because they lack the capacity for language; consequently, as beings with language, humans have a duty to speak on behalf of animals when their interests are threatened. While critical animal studies scholarship recognizes the “voicelessness” of animals “in the sense that their suffering is not heard by humans”, it also hears the voices of animals “as subjects who have desires, perspectives and interests” [49] (p. 497). Hence, the cry of a dog may be voiceless not because it cannot be heard auditorily, but because it fails to move the feelings of another.

Dogs can of course be highly vocal, and they do not need pre-recorded human voices to express themselves vocally. Canine voices include vocalizations that express inner states and communicate within the social environment [50]. Moreover, if “voice” as a concept is extended beyond the sonic, canine voice also includes a dog’s smells and behaviors including facial expressions, ear positions, and tail movement, among others, which may, in fact, carry greater significance among canines than just their vocalizations [51]. For dogs, then, voice might be better understood as a multimodal phenomenon mediated by a combination of sound and visual and olfactory signals.

Dogs that have learned to press buttons are gaining a novel human-like voice to add to their native canine voices. How does this alter their status in human societies?

Hunger and Devine, in a position aligned with that of some transhumanist thinkers who consider the technological enhancement of animals to be a moral human obligation, argue that humans should “uplift” nonhuman beings so that they can overcome their purported limitations [52]. Hunger and Devine’s breakthrough, as marketed to their followers, is in making Stella and Bunny’s thinking audible to humans. The limit to the dogs’ talking is only the number of buttons available to them.

However, although the soundboards promise to uplift Stella and Bunny from nonverbal communication to human language, their playback technology does not grant them fully fledged adult-human voices. Instead, Stella and Bunny communicate in ambiguous brief phrases that must constantly be interpreted for the viewer by Hunger and Devine. Claire Parkinson identifies a related trend of online videos that captures dogs vocalizing using their own vocal apparatus in similarly “broken” imitations of human language, often to express unconditional love for their owners. She argues that the infantilization of dogs as part of this anthropomorphic representation trades in a belittling cuteness of dogs [53]. Stella’s and Bunny’s “talking” is, likewise, cute in its infantile failure to produce flowing, grammatical sentences. This impression is reinforced by Bunny’s expectant stares towards her owner and the frequent childishness and banality of her observations. Devine’s videos, in particular, deliberately court consumers of cute canines. In her seminal theorization of cuteness, Sianne Ngai [54] observes the power of the cute object to infect the subject who perceives it, and Devine herself speaks to Bunny in strings of words lacking proper syntax, as if made cute by her encounters with her endearing companion. In these videos, Devine’s voice is, therefore, also not completely her own, finding itself pulled into a new orbit by the gravity of the voice she bestows on Bunny.

Yet, cuteness is not all that is offered here. Hunger and Devine also model what Miriam Lind [55] (p. 31), in analyzing the button-pressing interactions of women and dogs, calls “discussions”, which, she explains, involve “highly multimodal, multisensorial, situated cocreation of meaning” with dogs. The dogs may not understand what they are saying with the buttons in terms of human language (especially when used in combination); however, to then surmise that “proper” communication is not occurring (or that button pressing and its accompanying media are purely exploitative of dogs) is to miss how the back-and-forth between Hunger and Stella and Devine and Bunny during their button-pressing activities constitutes communication of responsiveness itself while simultaneously serving as a way to communicate basic needs, such as the desire for a walk. The dogs’ interests in maintaining the attention of their caregivers therefore intersects in button pressing with human fantasies of both the accessibility and childlike nature of dog minds.

Dog and human voices are also intermixed when dogs are anthropomorphized through the use of playback recordings, and human voices are zoomorphized through performances of “simplified” language, i.e., what Lind calls “delanguaging” [55] (p. 33). The appeal of a blurred boundary between these human and canine voices may be attributable to the apparent authenticity of the dog’s expression. In listening to the dogs press buttons, the audience of these videos enjoys the illusion of hearing dogs’ thoughts directly, but the dogs’ speech also invites active interpretation by Hunger and Devine, who attempt to make sense of its strange, infantile syntax (thereby maintaining the categorical hierarchy between the human’s and the nonhuman’s full and partial language capabilities, respectively). The buttons’ automated speech-like utterances do some of the work of translating canine utterances and give authenticity to the dogs’ speech because the meaningful utterance—passively listened to as the English language—is activated directly by the dog at the point of pressing the button. The source of the thought and the speech-like utterance, therefore, both appear to emerge from the dog as a thinking, intentional being as opposed to being split across a dog and a human mediator.

## 4. Conclusions

We summarize here the paradoxes that abound in contemporary soundboard-using dogs. Firstly, Hunger and Devine argue they are respecting canine uniqueness when “talking” with Stella and Bunny, respectively, but canine uniqueness appears to be privileged only through the paradoxical imposition of a human-designed communication technology. The world is familiar with these dogs through the very latest form of mass media—the short video shared through social media websites—and the dogs’ expressions are mediated by buttons onto which sounds can be recorded that only recently have become inexpensively available. As we have shown, these dogs are only the latest instantiation of a form of entertainment with a history of at least several centuries. People have been enjoying dogs (and other animals) performing trained human-like feats since at least the Middle Ages. These button-pressing dogs are presented as something more than entertainment, and data from their soundboard use have been provided to a research group who have published some peer-reviewed papers in scientific journals (e.g., [4,5]). Yet, most guardians of “talking dogs” reach their audiences via online social media, which are primarily sources of entertainment.

Secondly, these button-pressing dogs seem to offer unmediated access to their thoughts as the animals themselves activate buttons that articulate English words. However, paradoxically, the utterances that are expressed in this way are typically inchoate and require intensive interpretation from the always-present human to become coherent to an observer. Moreover, the dogs’ guardians present their dogs as spontaneously expressing themselves despite the many months of training required to bring them to do so. The short video format is unconducive to representing the process in full, thereby exacerbating this paradox, and the books by Hunger and Devine, while promising more insight, do not explore in any depth the relationship between a trained one-word expression of needs and a spontaneous conversation in sentence-like articulations between a canine and human guardian mediated by the buttons.

Thirdly, there is a paradox in believing that only the acquisition of a human skill, namely language, can serve as proof of the mental powers that predate the skill. This paradox is rooted in the erroneous assumption that while dogs possess rich internal mental states, they lack adequate channels to express them, which in turn stems from an anthropocentric worldview that privileges speech as the primary mode of communication and measures intelligence through skillful language use. Indeed, Hunger and Devine appear to have no qualms downplaying canine modes of expression and amplifying human speech despite the resemblances they draw across canine and human minds. This prompts the question: What effect does quieting canine vocality have on what the dogs’ guardians as well as viewers of their videos think they know about their dogs? When owners hear their dogs’ thoughts amplified as human voices, there is a risk that dog interests become self-evident; real dogs fade away, becoming what Donna Haraway [56] (p. 67) calls “humans in fur suits”, and the responsibility of remaining attentive to the alterity of a canine companion is forgotten. Dog vocalities—barks, howls, and whimpers —may help to remind us of our duty to remain curious about dog subjectivity and to be open to their living alterity as we communicate and share our lives with these animals.

## Data Availability

Data association with this article is available at: https://doi.org/10.58132/GZFKGO.
